# Blood-feeding patterns *of Culex pipiens* biotype *pipiens* and *pipiens/molestus* hybrids in relation to avian community composition in urban habitats

**DOI:** 10.1186/s13071-024-06186-9

**Published:** 2024-02-29

**Authors:** Rody Blom, Louie Krol, Melissa Langezaal, Maarten Schrama, Krijn B. Trimbos, Daan Wassenaar, Constantianus J. M. Koenraadt

**Affiliations:** 1 Laboratory of Entomology, Plant Sciences Group, Wageningen University & Research, Wageningen, The Netherlands; 2 Institute of Environmental Sciences, Leiden University, Leiden, The Netherlands; 3 Deltares, Utrecht, The Netherlands

**Keywords:** Mosquito, Blood meal, Host-feeding, Land use

## Abstract

**Background:**

*Culex pipiens* sensu stricto (s.s.) is considered the primary vector of Usutu virus and West Nile virus, and consists of two morphologically identical but behaviourally distinct biotypes (*Cx. pipiens* biotype *pipiens* and *Cx. pipiens* biotype *molestus)* and their hybrids. Both biotypes are expected to differ in their feeding behaviour, and *pipiens/molestus* hybrids are presumed to display intermediate feeding behaviour. However, the evidence for distinct feeding patterns is scarce, and to date no studies have related differences in feeding patterns to differences in host abundance.

**Methods:**

Mosquitoes were collected using CO_2_-baited traps. We collected blood-engorged *Cx. pipiens/torrentium* specimens from 12 contrasting urban sites, namely six city parks and six residential areas. Blood engorged *Cx. pipiens/torrentium* mosquitoes were identified to the species and biotype/hybrid level via real-time polymerase chain reaction (PCR). We performed blood meal analysis via PCR and Sanger sequencing. Additionally, avian host communities were surveyed via vocal sounds and/or visual observation.

**Results:**

We selected 64 blood-engorged *Cx. pipiens/torrentium* mosquitoes of which we successfully determined the host origin of 55 specimens. Of these, 38 belonged to biotype *pipiens*, 14 were *pipiens/molestus* hybrids and the identity of three specimens could not be determined. No blood-engorged biotype *molestus* or *Cx. torrentium* specimens were collected. We observed no differences in feeding patterns between biotype *pipiens* and *pipiens/molestus* hybrids across different habitats. Avian community composition differed between city parks and residential areas, whereas overall avian abundance did not differ between the two habitat types.

**Conclusions:**

Our results show the following: (1) *Cx. pipiens* s.s. feeding patterns did not differ between city parks and residential areas, regardless of whether individuals were identified as biotype *pipiens* or *pipiens/molestus* hybrids. (2) We detected differences in host availability between city parks and residential areas. (3) We show that in both urban habitat types, biotype *pipiens* and *pipiens/molestus* hybrids fed on both mammalian and avian hosts. This underscores the potential role in arbovirus transmission of biotype *pipiens* and *pipiens/molestus* hybrids.

**Graphical Abstract:**

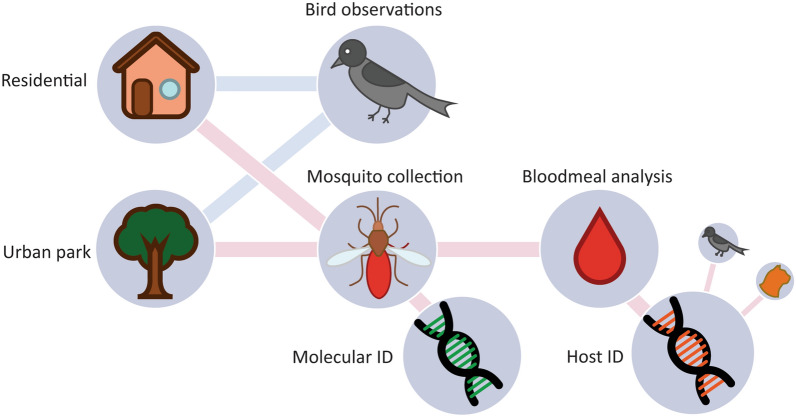

**Supplementary Information:**

The online version contains supplementary material available at 10.1186/s13071-024-06186-9.

## Background

Usutu virus (USUV) and West Nile virus (WNV) are two mosquito-borne flaviviruses that have recently emerged in North-Western Europe [1]. In the Netherlands, both USUV and WNV are closely monitored in mosquitoes and birds, which resulted in the first detection of virus circulation in 2016 and 2020, respectively [2–5]. In temperate regions, the ubiquitous mosquito species *Culex pipiens* sensu stricto (s.s.) is recognized as the primary enzootic vector of both USUV and WNV [6–9].

*Culex pipiens* s.s. is part of the *Culex pipiens* species complex. The complex comprises several species: *Cx. pipiens* s.s., *Cx. pallens*, *Cx. globocoxitus*, *Cx. australicus* and *Cx. quinquefasciatus* [10, 11]. *Culex pipiens* s.s. is the only member of the species complex to be present in the Netherlands. The sibling species *Cx. torrentium*, of which the female adults are nearly indistinguishable from *Cx. pipiens* s.s., also occurs in the Netherlands, albeit in low abundance [12].

*Culex pipiens* s.s. consists of two morphologically identical biotypes, namely *Cx. pipiens* biotype *pipiens* and *Cx. pipiens* biotype *molestus* [11, 13]. The two biotypes differ in their ecology, behaviour, and vector competence [7, 8]. Biotype *pipiens* mates in large, open spaces (eurygamy), whereas biotype *molestus* can mate in small, confined spaces (stenogamy). Additionally, biotype *molestus* can lay a first batch of eggs without taking a blood meal (autogeny), whereas biotype *pipiens* must take a blood meal in order to produce eggs (anautogeny). Until recently, in northern regions, populations of both biotypes were considered to be genetically isolated, as biotype *pipiens* was commonly associated with aboveground habitats, whereas biotype *molestus* was more associated with subterranean habitats [13, 14]. However, despite their ecological and behavioural differences, biotype *pipiens* and biotype *molestus* occur sympatrically aboveground in many European regions, and may interbreed to produce ‘hybrids’ [12, 15–17].

In order to understand the potential risk of WNV and USUV outbreaks, it is necessary to understand the host-feeding behaviour of the *Cx. pipiens* s.s. biotypes and their hybrids. Host-feeding is one of the primary determinants of vectorial capacity (the ability to transmit a pathogen), in addition to various other factors, such as vector competence [7, 18]. With regard to *Cx. pipiens* s.s., the paradigm is that both biotypes display distinct feeding behaviours. Following from laboratory choice experiments, biotype *pipiens* is often considered to be ornithophilic (preferring to feed on birds), whereas biotype *molestus* is considered to be mammophilic (preferring to feed on mammals) or even anthropophilic (preferring to feed on humans) [19–21]. Hybrids between the two biotypes are presumed to display intermediate host-feeding behaviour and may therefore act as bridge vectors [10, 14].

The host-feeding patterns of the *Cx. pipiens* s.s. biotypes and their hybrids remain enigmatic, as results from laboratory experiments and field studies often do not correlate. Myriad field studies have been published in which mosquito host-feeding patterns are described [10, 22–28]. However, in the majority of studies that include *Cx. pipiens* s.s. in the analysis, the distinction between the two *Cx. pipiens* s.s. biotypes and their hybrids is not made. Usually, all three are lumped together as ‘*Cx. pipiens’* or together with the sibling species *Cx. torrentium* as *Cx. pipiens/torrentium* [25, 29, 30]. Moreover, the few field studies in which a distinction between *Cx. pipiens* biotypes and hybrids was made, often did not demonstrate a pronounced difference in host-feeding patterns. This is most likely because host-feeding patterns are determined by both innate host preference and host availability [6, 31, 32]. Consequently, limited host availability can lead to two mosquito species or biotypes displaying similar host feeding patterns, despite their distinct innate host preferences. Host availability is strongly determined by habitat characteristics and seasonal variations in vertebrate communities. Consequently, differences in host-feeding patterns between different habitat types are expected. However, studies which compare host-feeding patterns in different habitat types remain scarce, often due to a lack of a sufficient number of blood-engorged specimens. For the Netherlands, no studies on the host-feeding patterns of mosquitoes, and *Cx. pipiens* s.s. in particular, have been published.

The objective of this study was to identify potential differences in feeding behaviours among the different *Cx. pipiens* s.s. biotypes in two contrasting urban habitat types. To this end, we conducted mosquito trapping in city parks and residential areas in order to collect blood-engorged mosquitoes and performed subsequent molecular blood meal analyses. In addition, we carried out inventories of the abundance and community composition of avian blood meal hosts.

## Methods

### Experimental design

**Fig. 1:**
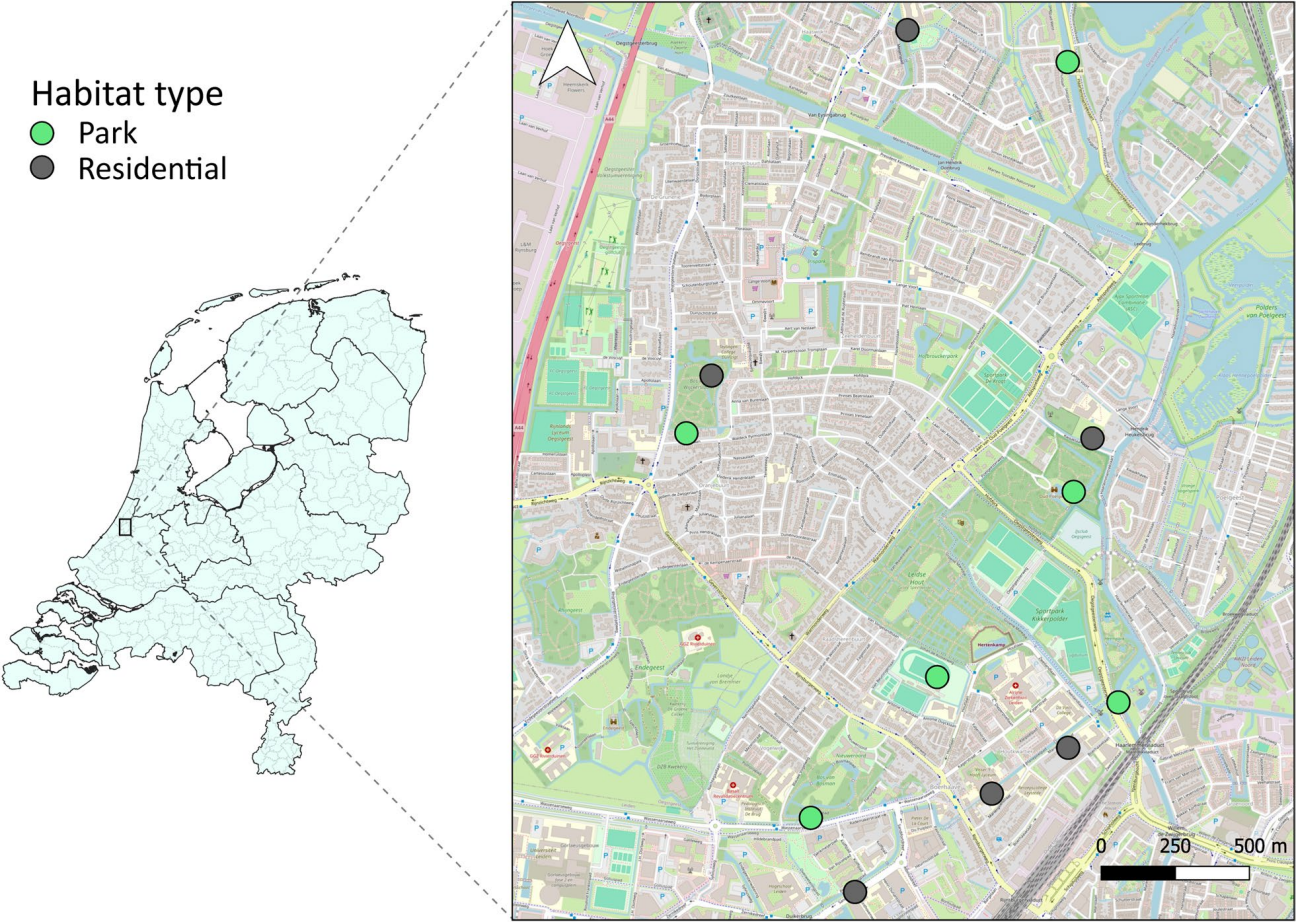
Map displaying the trapping sites in city parks and residential areas in the agglomeration of Leiden, the Netherlands. Three traps were placed at each site, with estimated distances between traps of 30–40 m

Mosquitoes were collected and avian hosts were inventoried in two contrasting urban habitats: city parks and residential areas. The study was conducted in the agglomeration of Leiden, the Netherlands. This urbanized area has a high human population density (5309.4/km^2^) and contains a large number of city parks. City parks differ strongly from the surrounding residential areas. City parks consist primarily of low, grassy vegetation and higher shrubs and trees, whereas residential areas consist primarily of impenetrable surfaces, such as buildings, roads and pavements. Mosquitoes were collected as described by Krol et al. [33]. In short, from the 30th of May until the 8th of July 2022, mosquitoes were trapped weekly for two nights per week at 12 sites (six city parks and six residential areas), using CO_2_-baited BG Pro (Biogents AG, Regensburg, Germany) traps (Fig. 1, Additional file 1: Table S1). Per site, three traps were placed in a triangular pattern, with approximately 30–40 m in between to limit interference between the traps. The maximum distance between two trap sites was approximately 3 km. To validate the difference between the two habitat types, the average percentage of imperviousness (impenetrability) of the ground surface was calculated per trap location. Imperviousness was derived from the Copernicus Imperviousness Density 2018 GIS layer, with a 10 × 10 m raster containing the percentage of imperviousness per cell [34]. Firstly, a centroid point was calculated between the three traps. Secondly, a 100 m distance buffer, based on the average flight distance of blood-engorged *Cx. pipiens* s.s. mosquitoes, was drawn around the centroid point [35, 36]. Lastly, within each buffer, the average imperviousness (sum of imperviousness/number of raster cells per buffer) was calculated (Additional file 1: Fig. S1). Spatial analyses were performed in QGIS (version 3.22 ‘Białowieża’) [37].

### Avian community composition

In order to identify whether mosquito feeding patterns differ due to host availability, avian hosts were surveyed in the trapping areas. Birds were surveyed during the same period of the year as mosquito sampling, to ensure that the observed species were available blood hosts during the time of the year when *Cx. pipiens* s.s. mosquitoes are active. Within hearing distance of each of the three mosquito traps per site, birds were counted in a point transect during 5-min intervals while walking along a standardized route between the traps [38]. The counts were performed six times at each of the sites, with the order of the site visits being randomized over two consecutive weeks. Counting started three times between 8:00 and 11:00 and three times between 15:00 and 18:00 (Central European Time). Data were collected by observing bird vocal sounds and/or through visual observation.

### Mosquito identification

Mosquitoes were identified morphologically to the species level following the identification key of Becker et al. [39]. Subsequently, blood-engorged (i.e. with a visible blood meal) *Cx. pipiens/torrentium* mosquitoes were selected for molecular species/biotype identification and blood meal analysis. Molecular species/biotype identification was performed following the DNA extraction and real-time polymerase chain reaction (PCR) protocol described by Vogels et al. [12], targeting the CQ11 microsatellite region. The fluorescent dyes of probes Cpp_Pip1 and Cpp_Pip2 were changed from VIC to Yakima Yellow (YAKYE), as VIC was no longer available from the manufacturer. Lab-reared biotype *pipiens* and biotype *molestus* were used as a positive control. In case the molecular assay did not result in a positive signal for biotype *pipiens*, biotype *molestus* or hybrids, an additional real-time PCR was performed for the identification of *Cx. torrentium,* using the *Cx. torrentium*-specific primer set and fluorescent probe as described by Vogels et al. [12]. For the *Cx. torrentium* identification assay, morphologically identified larval material was used as positive control. For both real-time PCR assays, nuclease-free water was used as a negative control. All real-time PCR reactions were performed on a CFX Opus qPCR system (Bio-Rad Laboratories, CA, USA). Mosquitoes of which the biotype or species could not be determined were excluded from further statistical analyses. In addition, in order to estimate the biotype composition per habitat type, 65 and 61 non-engorged *Cx. pipiens/torrentium* mosquitoes collected from parks and residential areas were selected, respectively. Species/biotype identification was performed with the real-time PCR protocol described above.

### Blood meal identification

Abdomens of blood-engorged mosquitoes were removed from the thorax using sterile forceps. Between samples, forceps were cleaned by dipping in 96% ethanol followed by flame sterilization. Subsequently, mosquito abdomens were placed individually in 2 ml Eppendorf tubes containing 4–6 zirconium oxide beads (0.5 mm), followed by dry homogenization (1 min, 400 Hz) and subsequent homogenization in 180 µl of ATL buffer (4 min, 400 Hz), using a TissueLyser II (Qiagen, Hilden, Germany). DNA extractions were performed using the DNeasy Blood & Tissue Kit (Qiagen, Hilden, Germany), following the manufacturer’s protocol. PCR runs were conducted using two sets of general vertebrate primers, in order to provide large coverage of vertebrate genomes. First, a primer set targeting a 358 bp sequence of the CytB region was used (forward: 5′-CCATCCAACATCTCAGCATGATGAAA-3′, reverse: 5′-CCCTCAGAATGATATTTGTCCTCA-3′) [40, 41]. In case of unsuccessful amplification with the primer pair targeting the CytB region, an additional PCR was performed with primers targeting a 244 bp sequence of the 16S ribosomal DNA (rDNA) region (forward: 5′-GCCTGTTTACCAAAAACATCAC-3′, reverse: 5′-GCCTGTTTACCAAAAACATCAC-3′) [42]. PCR reactions consisted of 12.5 µl MyTaq™ HS Red Mix (Meridian Bioscience, OH, USA), 0.8 µM of forward primer, 0.8 µM of reverse primer, 4.5 µl nuclease-free water and 4 µl of template DNA, adding up to a total volume of 25 µl. DNA from human (*Homo sapiens*) and chicken (*Gallus gallus*) blood was included as positive controls, and nuclease-free water was included in the reaction as non-template control. All PCR reactions were performed using a T100 Thermal Cycler (Bio-Rad Laboratories, CA, USA). PCR thermal cycling conditions were as follows: initial denaturation at 95 °C for 1 min, followed by 35 cycles of 95 °C for 15 s, 55 °C for 15 s, and 72 °C for 10 s. Product sizes of the amplicons were checked via visualization on a 1% agarose gel with 4 µl Midori Green (NIPPON Genetics EUROPE, GmbH, Düren, Germany). Successful amplifications were further analysed via Sanger sequencing, provided by an external sequencing service (Eurofins Genomics, Konstanz, Germany). PCR-negative samples were retested at least once. Samples selected for sequencing were prepared by combining 2.5 µl of forward primer, 2.5 µl of nuclease-free water and 5 µl of unpurified amplicon in test tubes provided by the external sequencing service. Sequences were trimmed in Geneious Prime 2023.0.4. Trimmed sequences were further analysed using a nucleotide Basic Local Alignment Search Tool (BLASTn) search against the National Center for Biotechnology (NCBI) GenBank database. The reference sequence with the highest pairwise identity (threshold ≥ 95%) was considered the host organism. Furthermore, sequence query cover and local presence of the species with the highest hit were taken into consideration when assigning a sequence to a host species.

### Statistical analyses

Imperviousness per habitat type was analysed using a Kruskal–Wallis test. For every bird species, the sum of the maximum number of individuals per site was used in further analyses, in order to correct for recounts. To test for differences in overall bird abundance between parks and residential areas, a Kruskal–Wallis test was conducted. In addition, we tested for differences in abundance of avian hosts that were detected in mosquito blood meals between both habitat types, excluding those that were not detected in blood meals, with a Kruskal–Wallis test. Differences in individual bird species abundance of species detected only in mosquito blood meals were tested with a Chi-square test with Bonferroni correction, followed by a Chi-square post hoc test. To test for differences in bird community composition between parks and residential areas, a Bray–Curtis distance matrix between the different data points was calculated and a non-metric multidimensional scaling (NMDS) analysis was run on the distance matrix. A permutational multivariate analysis of variance (PERMANOVA) was performed with the Bray–Curtis distances between sites as the response variable and parks versus residential areas as explanatory variable. Ratios between mammalian and avian blood meals were tested for all habitat type and biotype combinations using Pearson’s Chi-square test. Differences in the proportion of non-engorged biotype *pipiens*, biotype *molestus* and *pipiens/molestus* hybrids mosquitoes in city parks and residential areas were tested using Pearson’s Chi-square test. We used a significance level of 0.05 for all statistical tests. Statistical tests were performed in RStudio 2023.06.0 [43].

## Results

### Mosquito identification

In total, 10,277 adult female *Cx. pipiens/torrentium* mosquitoes were collected, of which 64 (0.62%) *Cx. pipiens/torrentium* were blood-engorged. Of the blood-engorged specimens, 31 were collected from city parks and 33 from residential areas. Out of 31 blood-engorged mosquitoes collected from city parks, 22 (71.0%) were identified as biotype *pipiens* and seven (22.6%) as *pipiens/molestus* hybrids*.* Molecular analysis of two specimens (6.5%) did not result in amplification. Out of 33 mosquitoes collected from residential areas, 24 (72.7%) were identified as biotype *pipiens* and 7 (21.2%) as *pipiens/molestus* hybrids. Here, molecular analysis of one specimen (3.0%) did not result in amplification. No blood engorged biotype *molestus* or *Cx. torrentium* were collected in either habitat type (Additional file 1: Table S2).

Of the non-engorged mosquitoes collected in city parks, 50 (76.9%) were identified as biotype *pipiens*, three (4.6%) were identified as biotype *molestus* and 10 (15.4%) were identified as *pipiens/molestus* hybrids. Of the non-engorged mosquitoes collected in residential areas, 44 (72.1%) were identified as biotype *pipiens*, 1 (1.6%) was identified as biotype *molestus* and 11 (18.0%) were identified as *pipiens/molestus* hybrids. No statistically significant differences in the proportion of biotype *pipiens*, biotype *molestus* and *pipiens/molestus* hybrids were detected between city parks and residential areas (*χ*^2^ = 1.0224, *df* = 2, *P* < 0.05) (Additional file 1: Fig. S2).

### Blood meal identification

Out of 64 samples, we successfully identified the blood meal host of 55 (86%) mosquitoes, from which 13 different host species were identified (Additional file 1: Table S2). All successful sequencing attempts were performed with primers targeting the CytB region. One sequence originating from biotype *pipiens* collected in a residential area was assigned to a pig (*Sus scrofa*) with a pairwise identity of 94.6%. Additionally, one sequence from biotype *pipiens* collected in a city park was assigned to the Eurasian blackbird (*Turdus merula*) with a pairwise identity of 94.2%. Both samples were excluded from further analyses. Other unsuccessful blood meal identifications were due to no amplification or failed sequencing attempts, resulting in double peaks or short, incomplete sequences. Unsuccessful host identification attempts were tried again with 16S primers, but this did not result in successful amplification of the target region. All unsuccessful host identification attempts were on blood meals collected from biotype *pipiens*.

**Fig. 2:**
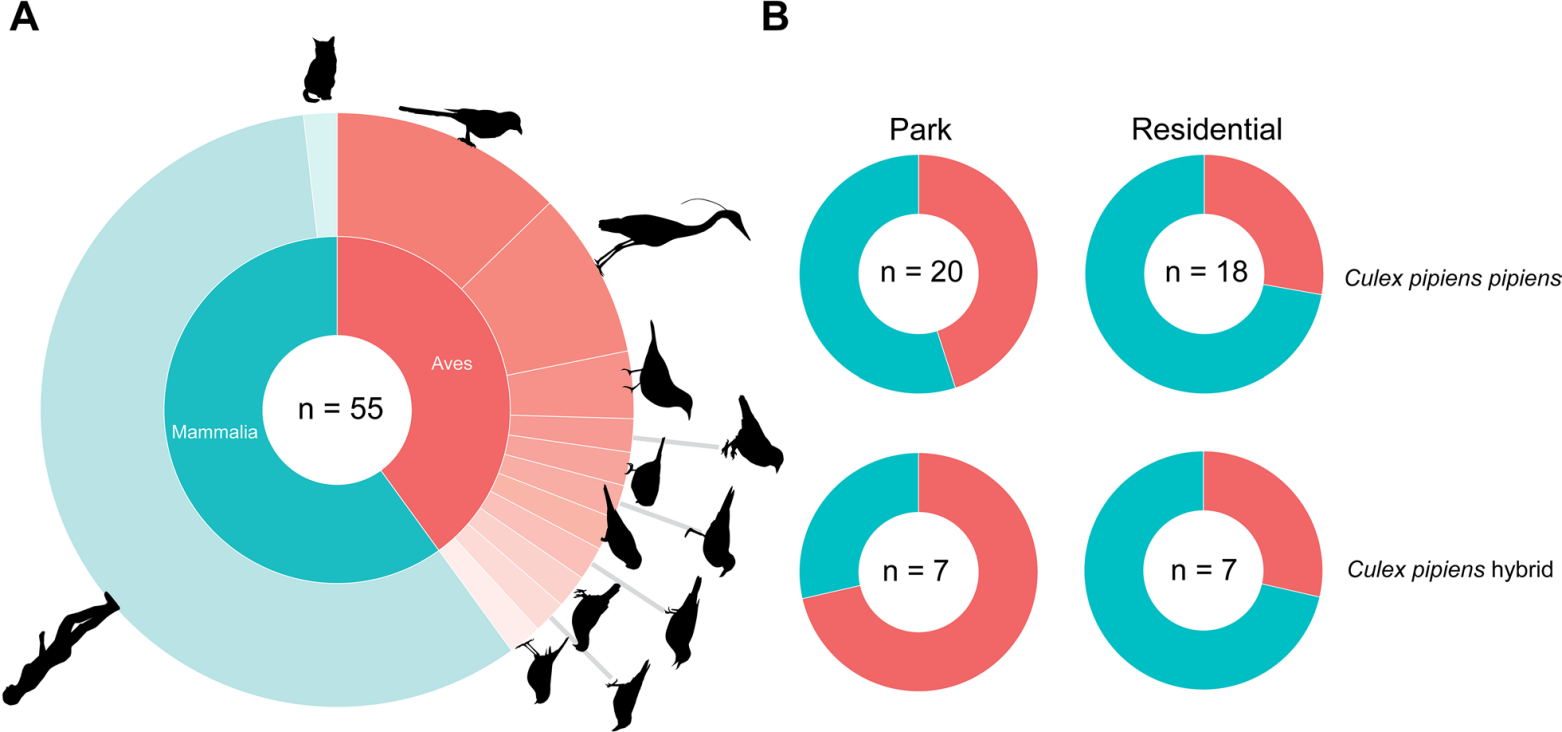
Donut-pie chart of all host species detected in mosquito blood meals, regardless of biotype (including three specimens that were not identified molecularly) and habitat (**A**). A full list of host species can be found in Additional file 1: Table S2. Blood meal origin of mammalian (blue) and avian (red) hosts per habitat, separated for biotype *pipiens* and *pipiens/molestus* hybrids (**B**). *Culex pipiens/torrentium* mosquitoes of which the species or biotype could not be determined were not included in (**B**)

In total, 31 (60%) blood meals taken by biotype *pipiens* and *pipiens/molestus* hybrids were from mammalian hosts, and 21 (40%) were taken from avian hosts (Fig. 2A). Furthermore, 29 of 55 (53%) successfully identified blood meals taken by *Cx. pipiens/torrentium* mosquitoes were collected in city parks, and 26 (47%) in residential areas. In city parks, 48% of blood meals from mosquitoes had a mammalian origin, whereas, from mosquitoes collected in residential areas, 72% of blood meals had a mammalian origin. When analysed by biotype, 55% of blood meals taken by biotype *pipiens* were of mammalian origin in city parks. Contrastingly, a lower percentage (29%) of blood meals taken by *pipiens/molestus* hybrids were of mammalian origin. In residential areas, 72% of blood meals from biotype *pipiens* and 71% of blood meals from *pipiens/molestus* hybrids were of mammalian origin. None of the above differences were significant following Pearson’s Chi-square tests (*P* > 0.05) (Fig. 2B). From six out of 12 locations (two parks, four residential areas), only human blood meals were collected, ranging between one and four human blood meals per location. One location (residential area) had a relatively high proportion of detected mammalian (six) versus avian (one) blood meals, and one location (residential area) had a relatively high proportion of detected avian (seven) versus mammalian (one) blood meals. There were no locations without mosquitoes that fed on mammalian hosts.

In total, 11 different avian host species were detected in mosquito blood meals, of which seven belong to the order of Passeriformes. Other avian hosts belong to the order of Charadriiformes (one), Columbiformes (one), Pelecaniformes (one) and Psittaciformes (one) (Additional file 1: Table S2). Only two blood meal host species (*Pica pica* and *Homo sapiens*) were detected in blood meals analysed from mosquitoes collected in both habitat types.

### Host availability

**Fig. 3:**
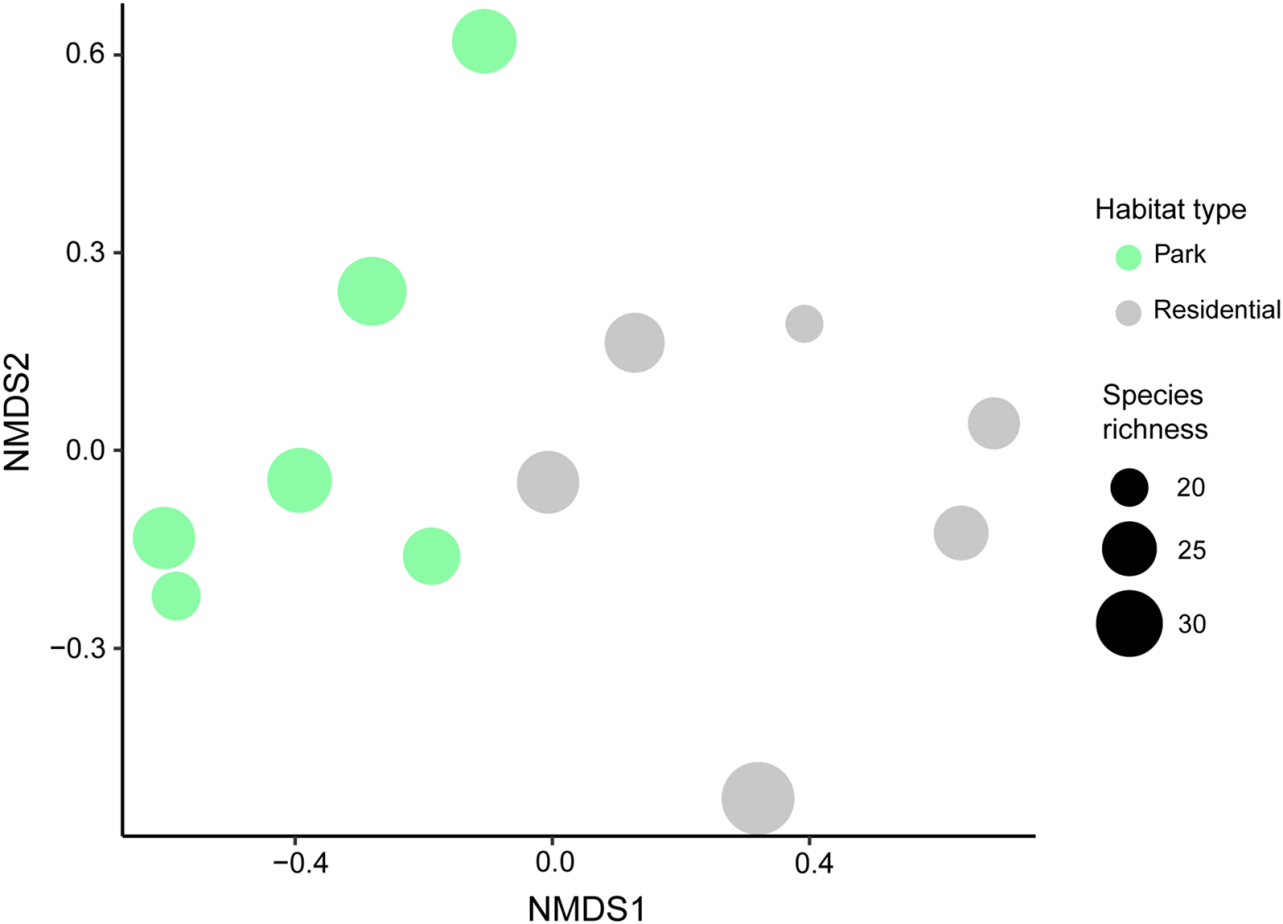
NMDS plot of avian hosts observed at city parks and residential areas. Each dot represents a park or residential sampling site. Dot size indicates the number of species per sampling site. Avian communities differed between the two habitat types (PERMANOVA, *R*^2^ = 0.20399, *df* = 1, *F* = 2.5626, *P* < 0.001)

**Fig. 4:**
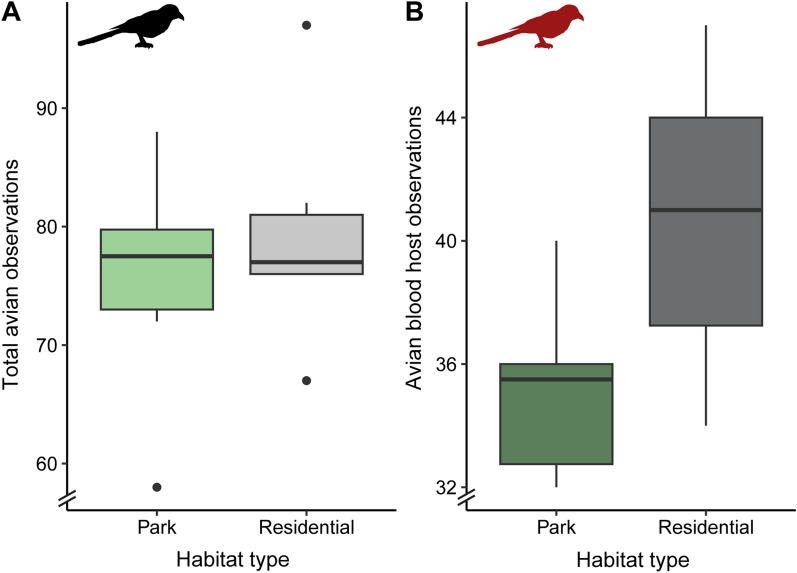
Boxplot of average number of maximum count per avian host species observed in city parks (green, *n* = 6) and residential areas (grey, *n* = 6) (**A**). Boxplot of average number of maximum count per avian host species observed in city parks (dark green, *n* = 6) and residential areas (dark grey, *n* = 6), with only avian hosts that were also detected in mosquito blood meals included (**B**)

In total, 929 birds (corrected for recounts) were observed representing 54 species over the course of 2 weeks (Additional file 1: Table S3). A statistically significant difference in overall bird community composition was found between the two habitat types (PERMANOVA, *R*^2^ = 0.20399, *df* = 1, *F* = 2.5626, *P* < 0.001) (Fig. 3). No statistically significant differences in overall bird abundance between residential areas and city parks were found (Kruskal–Wallis, *df* = 1, *P* > 0.05) (Fig. 4A). In addition, when only avian hosts that were detected in blood meals were analysed, no difference in abundance was detected (Kruskal–Wallis, *df* = 1, *P* > 0.05) (Fig. 4B). However, the abundance of two bird species differed significantly between the two habitat types (*χ*^2^ = 38.987, *df* = 12, *P* < 0.001). *Corvus monedula* (*P* < 0.01) was more abundant in residential areas, whereas *Turdus philomelos* (*P* < 0.05) was more abundant in city parks.

## Discussion

Here, we aimed to unravel potential differences in feeding patterns among different *Cx. pipiens* s.s. biotypes in two contrasting urban habitat types. We show that biotype *pipiens* and *pipiens/molestus* hybrids feed on a wide diversity of host species, even on a small spatial scale. Interestingly, in total, 60% of the blood meals taken by *Cx. pipiens* s.s. (regardless of biotype) were taken on mammalian hosts, almost exclusively on humans. In contrast, the number of different avian species found in blood meals was much higher. We did not find any statistically significant differences in the proportion of mammalian versus avian blood meals in different habitat types or between biotype *pipiens* and hybrids. However, we observed that the proportion of *pipiens/molestus* hybrids that fed on avian hosts in city parks was much higher compared to biotype *pipiens*. This is in contrast to our expectations, as *pipiens/molestus* hybrids are considered to be more mammophilic than biotype *pipiens* [21]. Overall, biotype *pipiens* fed slightly more on mammalian hosts than avian hosts, although no significant differences were found. In residential areas, the proportion of avian and mammalian hosts remained the same between biotype *pipiens* and *pipiens/molestus* hybrids. The absence of statistically significant differences between habitat types may be due to low statistical power, resulting from a low sample size. Blood-engorged mosquitoes are notoriously difficult to collect, as they are not attracted to CO_2_ post-blood-feeding. As an alternative or addition to CO_2_-baited traps, scientists can opt to use resting boxes, manual aspiration or citizen science [12, 23, 44, 45]. However, despite the lack of statistically significant differences in feeding patterns between biotypes and habitat types, the effect of habitat type seems large and in contrast to previous findings.

### Feeding patterns

Biotype-specific feeding patterns of *Cx. pipiens* s.s. remain enigmatic. Interestingly, outcomes of host preference studies performed under laboratory conditions differ strongly. In some cases, the distinction in biotype-specific host preferences is very pronounced [19], and in other cases, the biotype-specific differences are less clear [46]. Additionally, the findings of field studies on host-feeding patterns are also often conflicting. Osório et al. did observe distinct feeding patterns of both biotypes in the study they performed in urban and peri-urban habitats in Portugal [26]. Biotype *pipiens* fed primarily on birds, whereas biotype *molestus* fed mostly on human hosts. Interestingly, in studies by Martinez-de la Puente et al. and Gomes et al., both biotypes and *pipiens/molestus* hybrids fed more on avian hosts, without any marked differences between biotypes [27, 28]. In the study by Martinez-de la Puente et al. [28], mosquito collections were carried out in three contrasting habitats (natural, peri-urban and urban), and no differences in feeding patterns between the habitats were found. Gomes et al. [27] performed their collections mostly in semi-natural farming systems, but no comparison was made with a contrasting habitat type (such as an urban area). In all three aforementioned studies, host availability was not taken into account. In an extensive literature review by Brugman et al. on biotype-specific host-feeding patterns in the field, the data from the three studies were compiled, and the conclusion was drawn that all forms fed predominantly on birds and the observed differences between both biotypes and hybrids were minimal [6]. Contrastingly, in a recent field experiment conducted in a wide range of different habitats by Tiron et al. biotype *pipiens* fed primarily on avian hosts, whereas biotype *molestus* fed relatively more on mammals, including humans, which is in line with the general consensus [47]. No comparison in the feeding ratios was made between the habitat types.

### Role of avian species in arbovirus transmission

In our study, we show that both biotype *pipiens* and *pipiens/molestus* hybrids feed on avian and mammalian hosts in urban habitats. This highlights the species’ potential to transmit mosquito-borne viruses from competent reservoir hosts to humans. Several of the bird species detected in the blood meals analysed in our study are known to be susceptible to USUV and/or WNV infections, and some of the species may even develop disease symptoms. In particular, a large proportion of avian blood meals detected in our study originated from species within the order of Passeriformes. Of those, *T. philomelos* [48], *T. merula* [2], and *Erithacus rubecula* [49] have been reported to be susceptible to USUV infections. *Turdus philomelos* was found more in city parks than in residential areas. Two of the blood meals contained Eurasian blackbird (*T. merula*) DNA. After infection with USUV, *T. merula* may develop severe pathogenicity, including lesions which may eventually result in death [50]. Furthermore, a study from Spain highlighted the potential role of Passeriformes in USUV virus circulation, although the role of individual species was not studied [51]. *Pica pica* (Passeriformes) and *Psittacula krameri (*Psittaciformes*)* have been shown to be susceptible to WNV infections [52, 53]. Interestingly, *P. krameri* is an invasive species in North-Western Europe, which has its native range on the Indian subcontinent and in Africa. In the Netherlands, the amount of breeding pairs has increased strongly over the last three decades, in particular in the region where we conducted our study [54]. However, given its rapidly increasing abundance, it is important to study whether they produce enough viremia to become infectious and may therefore play a role in arbovirus amplification. In our study, five *Cx. pipiens* s.s. mosquitoes collected from city parks (three biotype *pipiens,* two *pipiens/molestus* hybrids) had fed on grey herons (*Ardea cinerea,* Pelecaniformes), a bird species from which WNV RNA was isolated in the province of Noord-Holland in the Netherlands in 2022 [55]. In an earlier study performed in the Camargue in the south of France, several bird species that were also found in blood meals in our study (*A. cinerea*, *Columba palumbus*, *Corvus corone*, *C. monedula*, *E. rubecula*, and *P. pica*) were identified as potential amplifying hosts [56]. However, for these species, the role they may play in amplifying WNV has not been studied experimentally, as has been done for several American bird species [57]. Therefore, even though earlier studies have highlighted the susceptibility of the aforementioned bird species to USUV and/or WNV infections, it remains unclear whether they play a role in the amplification, transmission and spread of these arboviruses.

### Effects of external factors on host-feeding patterns

Host-feeding patterns are strongly influenced by external factors, such as host availability [58, 59]. Host availability may fluctuate among different spatiotemporal contexts due to activity patterns and population dynamics of both mammalian and avian hosts. The latter occurs via natality, mortality and, in particular with regard to birds, migration [58, 60]. Furthermore, the behaviour of host species, such as roosting behaviour in birds, may affect vector–host contact rates [61, 62]. In addition, human host availability is strongly determined by climatological conditions and day length, as people may spend more time outdoors on warm summer nights. As reviewed by Fikrig and Harrington, the relative abundance of a particular host species in relation to all available hosts plays a crucial role in determining host-feeding patterns [58]. Therefore, it is important that mosquito host-feeding studies contain as much information on host availability as possible.

### Ecology of *Cx. pipiens* biotypes and hybrids

No blood-engorged biotype *molestus* were collected in our study, and the number of non-engorged biotype *molestus* mosquitoes was low. Biotype *molestus* specimens were collected in earlier studies from the Netherlands, albeit in relatively low numbers [12, 17]. Therefore, we expected to find more biotype *molestus* individuals in our study. Biotype *molestus* is commonly associated with urban environments, due to its stenogamy and presumed anthropophily [21]. However, the association of biotype *molestus* with urban habitats seems to differ among different ecological contexts, which is probably due to local climatological conditions and the availability of suitable mating and breeding sites [17, 28, 63]. Genetic misidentifications are unlikely, as the molecular marker we used to distinguish both biotypes and their hybrids (CQ11) is considered reliable, although several other options (such as COI, *ace*-2) are available [6, 63]. The low number of blood-engorged specimens can be partially explained by the fact that biotype *molestus* is known to be autogenous, meaning that they do not need a blood meal to produce a first batch of eggs. With regard to *Cx. pipiens* s.s. populations in Europe, the proportion of biotype *molestus* at northern latitudes is relatively low compared to biotype *pipiens,* as biotype *molestus* is less well adapted to unfavourable climatic conditions at northern latitudes due to its inability to enter diapause [16, 17]. In contrast, biotype *pipiens* is well adapted to colder temperatures at northern latitudes, enabling its survival in winter through diapause [64]. However, in winter, biotype *molestus* can be found indoors, where it remains actively blood-feeding, most likely on humans, thus enabling the survival of the population through winter [12].

Hybrids between biotype *pipiens* and biotype *molestus* are considered to play an important role as bridge vector, as they are presumed to display no preference towards either mammalian or avian hosts [10]. Consequently, it is expected that blood-engorged field-collected *pipiens/molestus* hybrids contain mammalian blood meals more often than those of the ornithophilic biotype *pipiens*. However, in our study and in the aforementioned studies, host-feeding patterns of *pipiens/molestus* hybrids seldom differ from biotype *pipiens* and/or biotype *molestus* collected from the same study areas. Furthermore, in many areas *pipiens/molestus* hybrids only occur in relatively low abundance, compared to biotype *pipiens* and/or biotype *molestus*, which is (with regard to biotype *pipiens*) in concordance with what we found in our study [16, 17, 27, 28]. With regard to WNV vector competence, peak transmission rates of biotype *molestus* and *pipiens/molestus* hybrids are lower than that of biotype *pipiens* [65]. Therefore, their role as bridge vectors may not be as large as often presumed. We hypothesize that in North-Western Europe, spillover of pathogens associated with *Cx. pipiens* s.s. primarily occurs via biotype *pipiens*, given its omnipresence, high abundance, WNV vector competence and host-feeding on both competent hosts and dead-end hosts.

## Conclusions

Here we provide insight into the host-feeding patterns of Dutch *Cx. pipiens* mosquitoes. We show that the feeding patterns of *Cx. pipiens* s.s. mosquitoes did not differ between habitat types, regardless of whether the specimens were identified as biotype *pipiens* or as *pipiens/molestus* hybrids. In both habitat types, there was an overlap in avian hosts as well as human hosts, highlighting the potential for mosquito-borne virus outbreaks in urban contexts. With regard to the transmission of USUV and WNV in urban habitats, additional studies on the relative proportion of avian and mammalian hosts in relation to host availability are necessary, especially in urban areas.

### Supplementary Information

**Additional file 1: Figure S1.** Mean percentage of imperviousness differed significantly between the two habitat types (Kruskal–Wallis, *df* = 1, *P* < 0.01). Average imperviousness of city parks was 4% versus 52% in residential areas. **Figure S2.** Relative biotype composition of non-engorged *Cx. pipiens* s.s. mosquitoes collected in city parks (*N* = 65) and residential areas (*N* = 61). No statistically significant differences in biotype/hybrid proportion were found between both habitat types (*χ*^2^ = 1.0224, *df* = 2, *P* > 0.05). **Table S1.** Overview of the coordinates of all trapping locations. Per trapping location, three traps were placed with an estimated 30–40 m distance in between. **Table S2.** Blood meal origin of *Cx. pipiens* pipiens and pipiens/molestus hybrids collected in city parks and residential areas. **Table S3.** Total number of observations per bird species per habitat type (city parks and residential areas). The number of observations per species is not corrected for recounts.

## Data Availability

All data are available upon request to the corresponding author.
