# Supplementary material for: Blood-feeding patterns of Culex pipiens biotype pipiens and pipiens/molestus hybrids in relation to avian community composition in urban habitats
Source: Parasit Vectors. 2024 Feb 29;17:95. doi: 10.1186/s13071-024-06186-9 (PMC10902945; doi:10.1186/s13071-024-06186-9)
Supplement: Supplementary file 1 — Additional file 1: Figure S1. Mean percentage of imperviousness differed significantly between the two habitat types (Kruskal–Wallis, df = 1, P < 0.01). Average imperviousness of city parks was 4% versus 52% in residential areas. Figure S2. Relative biotype composition of non-engorged Cx. pipiens s.s. mosquitoes collected in city parks (N = 65) and residential areas (N = 61). No statistically significant differences in biotype/hybrid proportion were found between both habitat types (χ2 = 1.0224, df = 2, P > 0.05). Table S1. Overview of the coordinates of all trapping locations. Per trapping location, three traps were placed with an estimated 30–40 m distance in between. Table S2. Blood meal origin of Cx. pipiens pipiens and pipiens/molestus hybrids collected in city parks and residential areas. Table S3. Total number of observations per bird species per habitat type (city parks and residential areas). The number of observations per species is not corrected for recounts. [file 13071_2024_6186_MOESM1_ESM.docx]

**Additional file**

**
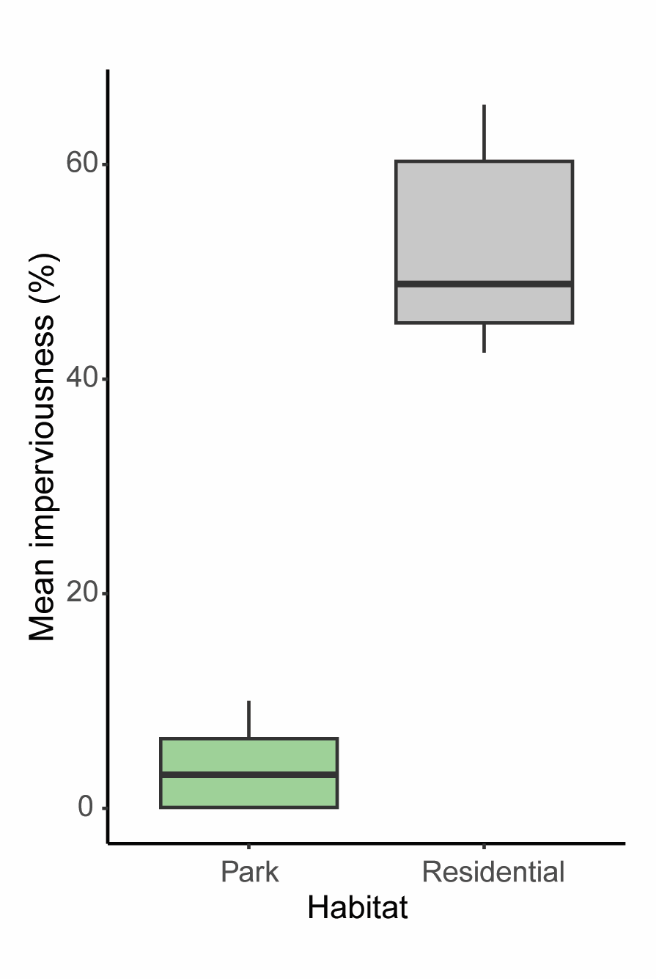
**

**Figure S1:** Mean percentage of imperviousness differed significantly between the two habitat types (Kruskal-Wallis, df = 1, p < 0.01). Average imperviousness of city parks was 4% versus 52% in residential areas.


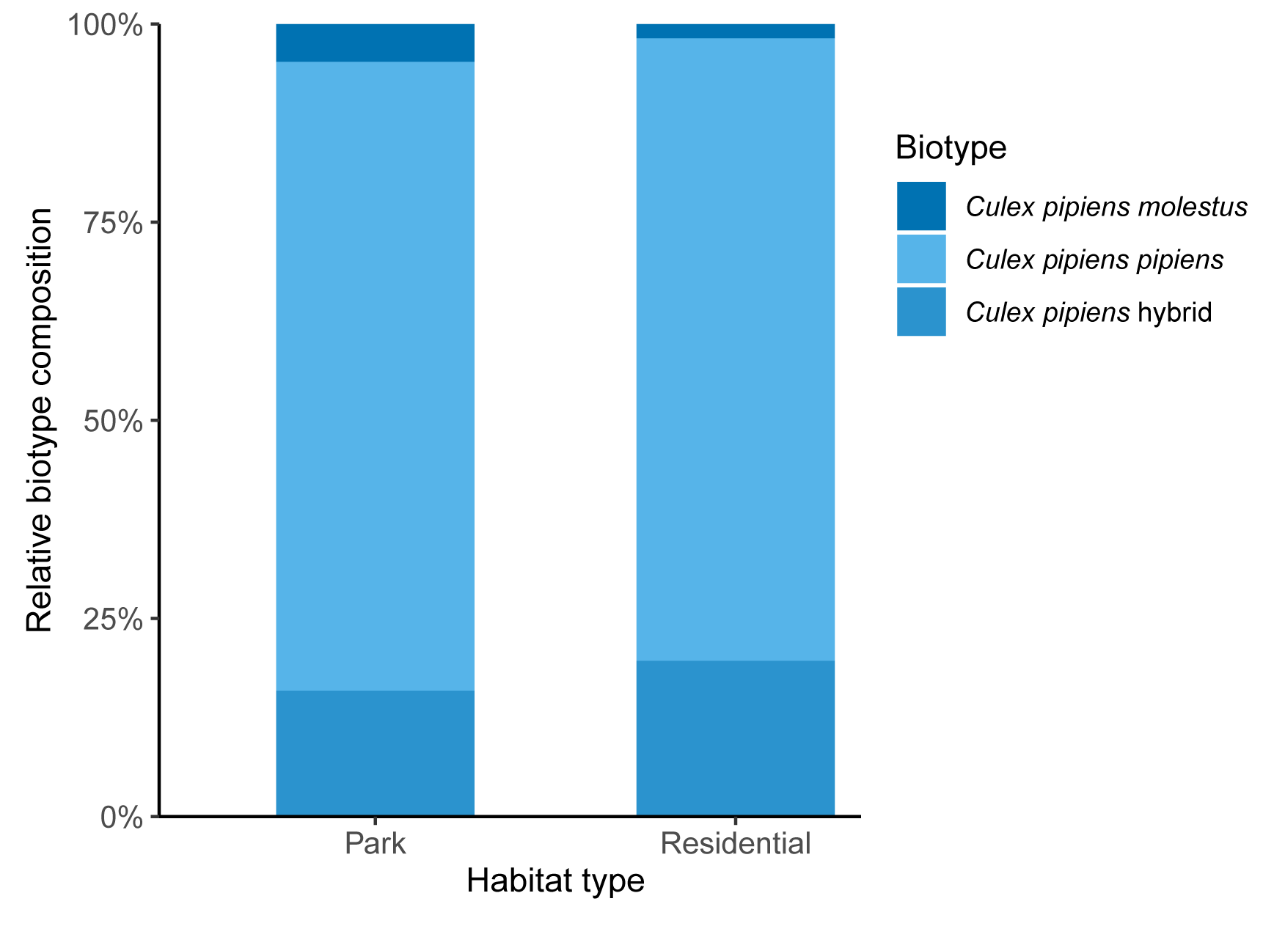


**Figure S2.** Relative biotype composition of non-engorged *Cx. pipiens* s.s. mosquitoes collected in city parks (N=65) and residential areas (N=61). No statistically significant differences in biotype/hybrid proportion were found between both habitat types (χ^2^ = 1.0224, df = 2, *Ρ* > 0.05).

**Table S1.** Overview of the coordinates of all trapping locations. Per trapping location, three traps were placed with an estimated 30–40m distance in between.

| **Habitat type** | **Latitude** | **Longitude** |
| --- | --- | --- |
| Park | 52.17069 | 4.473202 |
|  | 52.17419 | 4.488376 |
|  | 52.19356 | 4.48587 |
|  | 52.17496 | 4.479434 |
|  | 52.18056 | 4.486168 |
|  | 52.18233 | 4.467063 |
| Residential | 52.19453 | 4.477967 |
|  | 52.17282 | 4.485896 |
|  | 52.18407 | 4.468306 |
|  | 52.16845 | 4.475381 |
|  | 52.17143 | 4.482129 |
|  | 52.18217 | 4.487094 |

**Table S2.** Bloodmeal origin of *Cx. pipiens pipiens* and *pipiens/molestus* hybrids collected in city parks and residential areas.

|  |  | **City park** |  | **Residential** |  |
| --- | --- | --- | --- | --- | --- |

| Host class | Host order | Host species | Biotype *pipiens* | *pipiens/molestus* hybrid | *NA* | Biotype *pipiens* | *pipiens/molestus* hybrid | *NA* |
| --- | --- | --- | --- | --- | --- | --- | --- | --- |

| Mammalia |  | *Felis catus* |  |  |  | 1 |  |  |
| --- | --- | --- | --- | --- | --- | --- | --- | --- |
|  |  | *Homo sapiens* | 11 | 2 | 2 | 12 | 5 |  |
| Aves | Passeriformes | *Corvus corone* | 1 |  |  |  |  |  |
|  |  | *Corus monedula* |  |  |  | 1 |  |  |
|  |  | *Cyanistes caureleus* |  |  |  | 1 |  |  |
|  |  | *Erithacus rubecula* | 1 |  |  |  |  |  |
|  |  | *Pica pica* | 3 | 1 |  | 1 | 1 | 1 |
|  |  | *Turdus merula* | 1 | 1 |  |  |  |  |
|  |  | *Turdus philomelos* |  | 1 |  |  |  |  |
|  | Charadriformes | *Larus spp.* |  |  |  | 1 |  |  |
|  | Columbiformes | *Columba palumbus* |  |  |  |  | 1 |  |
|  | Pelecaniformes | *Ardea cinerea* | 3 | 2 |  |  |  |  |
|  | Psittaciformes | *Psittacula krameri* |  |  |  |  |  | 1 |

**Table S3.** Total number of observations per bird species per habitat type (city parks and residential areas). The number of observations per species is not corrected for recounts.

|  | **Park** | **Residential** | **Total** |
| --- | --- | --- | --- |
| **Anseriformes** | **88** | **27** | **115** |
| *Alopochen aegyptiaca* | 49 | 2 | 51 |
| *Anas platyrhynchos* | 32 | 24 | 56 |
| *Anser anser* | 2 | 0 | 2 |
| *Branta bernicla* | 1 | 0 | 1 |
| *Branta canadensis* | 0 | 1 | 1 |
| *Cygnus olor* | 4 | 0 | 4 |
| **Caprimulgiformes** | **33** | **66** | **99** |
| *Apus apus* | 33 | 66 | 99 |
| **Charadriiformes** | **104** | **326** | **430** |
| *Haematopus ostralegus* | 4 | 12 | 16 |
| *Larus argentatus* | 22 | 70 | 92 |
| *Larus fuscus* | 48 | 225 | 273 |
| *Larus ridibundus* | 26 | 17 | 43 |
| *Sterna hirundo* | 4 | 2 | 6 |
| **Ciconiiformes** | **1** | **1** | **2** |
| *Ciconia ciconia* | 1 | 1 | 2 |
| **Columbiformes** | **90** | **97** | **187** |
| *Columba livia* | 0 | 18 | 18 |
| *Columba oenas* | 1 | 0 | 1 |
| *Columba palumbus* | 85 | 50 | 135 |
| *Streptopelia decaocto* | 4 | 29 | 33 |
| **Coraciiformes** | **1** | **0** | **1** |
| *Alcedo atthis* | 1 | 0 | 1 |
| **Galliformes** | **0** | **75** | **75** |
| *Gallus gallus domesticus* | 0 | 75 | 75 |
| **Gruiformes** | **111** | **34** | **145** |
| *Fulica atra* | 96 | 24 | 120 |
| *Gallinula chloropus* | 15 | 10 | 25 |
| **Passeriformes** | **1171** | **957** | **2128** |
| *Acrocephalus scirpaceus* | 4 | 0 | 4 |
| *Carduelis carduelis* | 0 | 2 | 2 |
| *Certhia brachydactyla* | 69 | 20 | 89 |
| *Corvus corone* | 35 | 29 | 64 |
| *Corvus monedula* | 115 | 379 | 494 |
| *Cyanistes caeruleus* | 34 | 37 | 71 |
| *Erithacus rubecula* | 86 | 39 | 125 |
| *Fringilla coelebs* | 51 | 10 | 61 |
| *Garrulus glandarius* | 19 | 2 | 21 |
| *Muscicapa striata* | 3 | 0 | 3 |
| *Parus major* | 129 | 61 | 190 |
| *Passer domesticus* | 4 | 36 | 40 |
| *Passer montanus* | 1 | 1 | 2 |
| *Phylloscopus collybita* | 65 | 25 | 90 |
| *Pica pica* | 54 | 83 | 137 |
| *Prunella modularis* | 4 | 2 | 6 |
| *Sitta europaea* | 19 | 3 | 22 |
| *Sturnus vulgaris* | 1 | 5 | 6 |
| *Sylvia atricapilla* | 26 | 7 | 33 |
| *Sylvia borin* | 2 | 0 | 2 |
| *Taeniopygia castanotis* | 0 | 61 | 61 |
| *Troglodytes troglodytes* | 163 | 42 | 205 |
| *Turdus merula* | 243 | 106 | 349 |
| *Turdus philomelos* | 44 | 7 | 51 |
| **Pelecaniformes** | **10** | **5** | **15** |
| *Ardea cinerea* | 10 | 5 | 15 |
| **Piciformes** | **19** | **3** | **22** |
| *Dendrocopos major* | 16 | 3 | 19 |
| *Dryobates minor* | 3 | 0 | 3 |
| **Podicipediformes** | **20** | **2** | **22** |
| *Podiceps cristatus* | 20 | 2 | 22 |
| **Psittaciformes** | **109** | **100** | **209** |
| *Agapornis sp.* | 0 | 12 | 12 |
| *Melopsittacus undulatus* | 0 | 8 | 8 |
| *Nymphicus hollandicus* | 0 | 12 | 12 |
| *Psittacula krameri* | 109 | 68 | 177 |
| **Suliformes** | **1** | **1** | **2** |
| *Phalacrocorax carbo* | 1 | 1 | 2 |
| **Total** | **1758** | **1694** | **3452** |
